# Serum albumin predicts survival in patients with hilar cholangiocarcinoma

**DOI:** 10.1093/gastro/gow021

**Published:** 2016-07-06

**Authors:** Abhijeet Waghray, Anastasia Sobotka, Carlos Romero Marrero, Bassam Estfan, Federico Aucejo, KV Narayanan Menon

**Affiliations:** 1 Department of Medicine*,* MetroHealth Medical Center*,* Cleveland*,* OH*,* USA; 2 Department of Gastroenterology and Hepatology*,* Cleveland Clinic Foundation*,* Cleveland*,* OH*,* USA; 3 Department of Hepatobiliary and Transplant Surgery*,* Cleveland Clinic Foundation*,* Cleveland*,* OH*,* USA

**Keywords:** hilar cholangiocarcinoma, prognostic factors, survival, albumin

## Abstract

**Background and aims:** Hilar cholangiocarcinoma is a devastating malignancy with incidence varying by geography and other risk factors. Rapid progression of disease and delays in diagnosis restrict the number of patients eligible for curative therapy. The objective of this study was to determine prognostic factors of overall survival in all patients presenting with hilar cholangiocarcinoma.

**Methods:** All adult patients with histologically confirmed hilar cholangiocarcinoma from 2003 to 2013 were evaluated for predictors of survival using demographic factors*,* laboratory data*,* symptoms and radiological characteristics at presentation.

**Results:** A total of 116 patients were identified to have pathological diagnosis of hilar cholangiocarcinoma and were included in the analysis. Patients with a serum albumin level >3.0 g/dL (*P < *0.01)*,* cancer antigen 19‐9 ≤200 U/mL (*P = *0.03)*,* carcinoembryonic antigen ≤10 ìg/L (*P < *0.01) or patients without a history of cirrhosis (*P < *0.01) or diabetes (*P = *0.02) were associated with a greater length of overall survival. A serum albumin level >3.0 g/dL was identified as an independent predictor of overall survival (hazard ratio 0.31; 95% confidence interval 0.14–0.70) with a survival benefit of 44 weeks.

**Conclusion:** This study was the largest analysis to date of prognostic factors in patients with hilar cholangiocarcinoma. A serum albumin level >3.0 g/dL conferred an independent survival advantage with a significantly greater length of survival.

## Introduction

Cholangiocarcinoma is the second most common primary hepatic malignancy worldwide, eclipsed only by hepatocellular carcinoma [1]. Hilar cholangiocarcinoma—or Klatskin’s tumor—was first described in 1965 and accounts for 40–60% of all cholangiocarcinomas [2–5]. A rare entity in the United States*,* hilar cholangiocarcinoma is a devastating malignancy that carries a poor prognosis. Its incidence in Asia is as high as 71 per 100 000 men and 31 per 100 000 women. Patients are affected in the sixth or seventh decade of life, with presentation before the age of 40 being a rarity [6]. Incidence varies by geography and other risk factors, such as advanced age (greater than 65)*,* male gender*,* cirrhosis*,* parasitic liver disease*,* inflammatory bowel disease*,* chronic pancreatitis*,* biliary disease (cysts and stones), and primary sclerosing cholangitis [1*,* 7*,* 8].

Early symptoms for hilar cholangiocarcinoma are non-specific. Abdominal pain*,* pruritus*,* weight loss*,* fatigue*,* dark urine/clay-colored stool are common [9–11]. As this malignancy affects the bifurcation of the common hepatic biliary duct*,* unilateral hepatic duct obstruction may not present as overt jaundice until later in the course of the disease. The diagnosis of hilar cholangiocarcinoma remains challenging, as masses are small at clinical presentation and not frequently visualized on computed tomography (CT) scans or magnetic resonance imaging (MRI). Attempts at pathological diagnosis via endoscopic procedures often yields inconclusive results, even after multiple attempts (ranges from 44–80%) [12*,* 13]. Surgical resection and liver transplantation are curative options for hilar cholangiocarcinoma but require early intervention [14]; further*,* surgical resection is technically challenging, given the tumor's proximity to critical vascular structures and the need for adequate surgical margins [15]. Only a select few can be offered liver transplantation [16*,* 17]. Rapid progression of disease and delays in diagnosis further hamper efforts at curative therapy. Despite a paucity of data*,* chemotherapy may be indicated in patients with adequate functional status and unresectable disease; unfortunately*,* many patients are limited to palliative measures, including percutaneous transhepatic biliary drainage*,* biliary stents, or palliative bypass surgery. In recent years*,* tumor markers such as cancer antigen (CA) 19‐9 and carcinoembryonic antigen (CEA) have shown promise for diagnosing and monitoring treatment of hilar cholangiocarcinoma. When combined with other diagnostic modalities, they have a sensitivity of 89% and specificity of 86% [18].

Understanding factors that determine prognosis is important for improving outcomes and allowing clinicians to stratify patients for treatment; unfortunately*,* data remain limited. The few studies completed have assessed prognostic factors in surgical patients only and no study to date has provided a comprehensive evaluation of prognostic variables in all patients presenting with hilar cholangiocarcinoma. This study assessed presenting laboratory values*,* demographics*,* and medical history (e.g. risk factors for cholangiocarcinoma) to determine prognostic indicators in all patients presenting with hilar cholangiocarcinoma.

## Methods

All adult patients with hilar cholangiocarcinoma, pathologically confirmed between September 2003 and September 2013 at the Cleveland Clinic Foundation, were retrospectively identified and included in the analysis. Diagnosis was confirmed histologically by bile duct brushings or biopsies. Patients included those referred to the Cleveland Clinic Foundation for further evaluation or those initially evaluated at our institution. The following data were collected for all patients: demographic data (gender*,* race and age)*,* laboratory data (total bilirubin, alkaline phosphatase*,* serum albumin*,* CEA, CA19‐9)*,* symptoms prior to presentation*,* medical history [diabetes*,* cirrhosis, primary sclerosing cholangitis (PSC)]*,* therapeutic interventions and overall survival. The presence of cirrhosis was determined by laboratory and radiographical evidence when biopsy evidence was not available. All laboratory data were collected at the time of initial presentation.

The treatment modality received by individual patients was determined by a multidisciplinary treatment team, with final therapy completed at the patient's discretion. Primary operative management was hepatic resection. Patients not eligible for surgical resection were considered for liver transplantation. After liver transplantation, a standardized immunosuppression regimen was followed. The primary endpoint was overall survival, defined as the time from initial symptom onset until the date of death/survival. This study was approved by the Cleveland Clinic's Institutional Review Board.

## Statistical Analysis

Univariate and multivariate analysis of demographic data (age*,* gender*,* and race)*,* risk factors for cholangiocarcinoma (cirrhosis*,* smoking/alcohol history*,* diabetes*,* and primary sclerosing cholangitis)*,* presenting laboratory data (total bilirubin*,* alkaline phosphatase*,* serum albumin*,* CEA and CA19‐9) were evaluated. Patients with incomplete data were excluded (those with less than 20% of variables defined) or if current status (alive/dead) could not be verified. Categorical data were compared with Fisher’s exact tests and quantitative variables were represented as mean with standard error. Overall survival was described using Kaplan-Meier estimates, with overall survival determined by survival curves. Multivariate analysis was performed using the Cox proportional hazards model. Overall survival was defined as time from start of symptoms to time of death with patients alive at the end of the study censored and was represented as median with 95% confidence interval (CI). Sub-group analysis of patients who underwent surgical resection was completed. A *P-*value <0.05 was considered significant. Data were analysed using JMP version 9.0 (SAS Institute Inc.*,* Cary*,* NC*,* USA).

## Results

One hundred and twenty-four patients with pathologically diagnosed hilar cholangiocarcinoma were identified. Eight patients were excluded for incomplete data. Complete demographic and clinical data are shown in Table 1

**Table 1.** Demographics of hilar cholangiocarcinoma patients

Parameter	Number of patients (*n* = 116)
Gender*, n* (%)	
Male	76 (65.5)
Female	40 (34.5)
Age at diagnosis*,* years	67.3 ± 1.2
Body mass index*,* kg/m^2^	27.1 ± 0.7
Race*, n* (%)	
Caucasian	92 (79.3)
African American	9 (7.8)
Asian	5 (4.3)
Unknown/not reported	10 (8.6)
Symptom*, n* (%)	
Jaundice	76 (65.5)
Weight loss	53 (45.7)
Fatigue	32 (27.6)
Abdominal pain	47 (40.5)
Nausea	18 (15.5)
Decreased appetite	27 (23.3)
Pruritus	32 (27.6)
Pale stool	28 (24.1)
Dark urine	39 (33.6)
Social history*, n* (%)	
Smokers	53 (48.2)
Alcohol use	42 (37.8)

Data presented as *n*  (%) and mean ± standard deviation

. Sixty-six percent were male and 85% were caucasian. The average age of presentation was 67.3 ± 1.2 years. The predominant presenting symptom was jaundice in 65.5% patients. Weight loss was the second most common symptom at presentation (45.7%; mean 23.8 ± 2.1 pounds lost at presentation). On average, patients presented with 3.2 ± 0.2 symptoms. A history of tobacco use was present in 48.2% patients and 37.8% had a history of alcohol use. Diabetes was present in 27.2% patients. Median overall survival was 37.8 ± 4.1 weeks.

On univariate analysis of variables at patient presentation*,* predictors of overall survival included albumin >3.0 g/dL*,* CA19‐9 ≤ 200 U/mL*,* CEA ≤ 10 μg/L and patients without a history of cirrhosis or diabetes (Table 2

**Table 2.** Univariate analysis of parameters on patient presentation

Parameter	Survival time*,* days*, *median (95% CI)	*P-*value
Age*,* years		0.40
≤65	271 (221–409)	
>65	255 (191–334)	
Gender		0.56
Male	254.5 (204–344)	
Female	274.5 (194–337)	
Race		0.62
Caucasian	257.5 (210–333)	
African American	380 (55–670)	
Asian	409 (68–487)	
Unknown/not reported	170 (123–217)	
Albumin*,* g/dL		<0.01
>3.0	467 (122–194)	
≤3.0	155 (257–572)	
Cancer antigen 19‐9*,* U/mL		0.03
≤200	444.5 (210–532)	
>200	203 (142–238)	
Carcinoembryonic antigen*,* μg/L		<0.01
≤10	305.5 (221–469)	
>10	188 (84–210)	
Alkaline phosphatase*,* U/L		0.08
≤400	210 (122–278)	
>400	271 (191–409)	
Total bilirubin*,* μmol/L		0.77
≤10	271*	
>10	241.5*	
Cirrhosis		<0.01
Yes	110 (8–155)	
No	271 (221–337)	
Diabetes		0.02
Yes	225 (122–326)	
No	271 (221–366)	
Primary sclerosis cholangitis		0.78
Yes	188^a^	
No	271 (212–336)	
Smoking		0.97
Yes	258 (204–352)	
No	265 (191–334)	
Alcohol use		0.80
Yes	255 (200–380)	
No	265 (207–336)	
Weight loss*,* pounds		0.53
>20	365 (203–409)	
≤20	352 (204–469)	

^a^Patient still alive at time of analysis*,* thus upper limit of 95% CI not determined.

). On multivariate analysis serum albumin >3.0 g/dL was identified as the only independent predictor of overall survival (*P < *0.01; hazard ratio (HR) 0.31; 95% CI 0.14–0.70).

**Figure 1.**
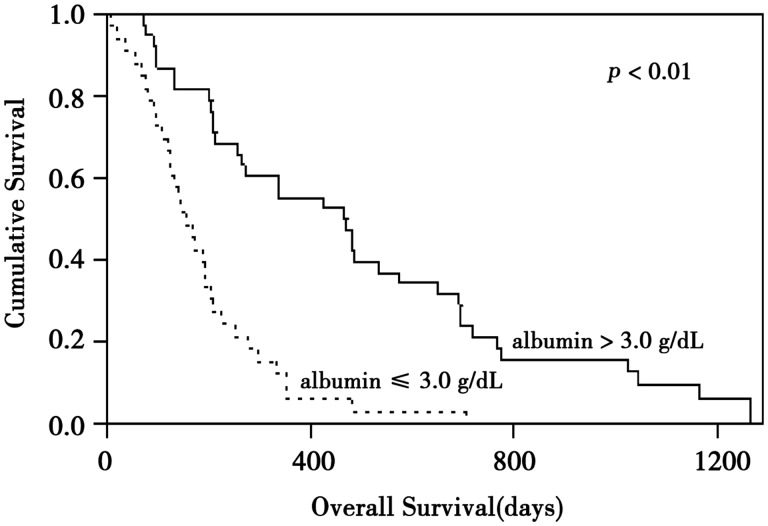
Kaplan-Meier analysis of overall survival in patients with hilar cholangiocarcinoma and an albumin >3.0 g/dL *vs .*≤3.0 g/dL.

**Figure 2.**
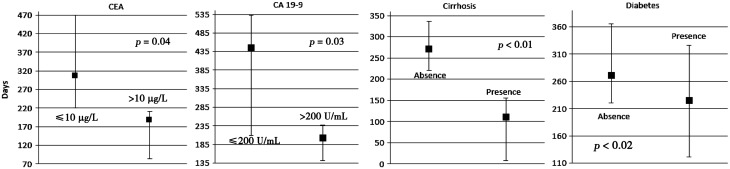
Overall survival [median days (95% CI)] in patients with hilar cholangiocarcinoma and (1) CEA level: ≤10μg/L *vs.* >10 μg/L; (2) CA19‐9: ≤200 U/mL *vs.* >200 U/mL; (3) Cirrhosis: absence *vs.* presence; (4) Diabetes: absence *vs.* presence.

For patients with a serum albumin >3.0 g/dL*,* the median survival period was 467 days (95% CI 257–572), which is significantly longer than 155 days (95% CI 122–194) in patients with an albumin ≤3.0 g/dL (*P < *0.01; Figure 1

). In the latter group*,* concomitant cirrhosis was present in 18.2% of patients. For patients with a CEA > 10 μg/L*,* the median survival was 188 days (95% CI 84–210), compared with 305.5 days (95% CI 221–469) in patients with CEA ≤ 10 μg/L (*P < *0.01). Patients with CA19‐9 ≤ 200 U/mL had a median survival period of 444.5 days (95% CI 210–532), compared with 203 days (95% CI 142–238) for those with a value greater than 200 U/mL (*P = *0.03). In patients with a history or cirrhosis*,* overall survival was significantly shorter [110 days (95% CI 8–155) *vs.* 271 days (95% CI 221–337); *P < *0.01]. Lastly, patients with diabetes experienced a shorter overall survival [225 days (95% CI 122–326)] than those without diabetes [271 (95% CI 221–366) days; *P = *0.02; Figure 2

].

Patients were treated with surgical resection (16.4%; *n = *19)*,* liver transplantation (0.9%; *n = *1)*,* brachytherapy (3.4%; *n = *4)*,* radiation (7.8%; *n = *9)*,* chemotherapy (22.4%; *n = *26)*,* biliary stenting (82.8%; *n = *96) and percutaneous transhepatic biliary catheters (69.8%; *n = *81). Patients with surgical resection or liver transplantation had an overall survival greater than those who were not surgical candidates [708 days (95% CI 366–1023) *vs.* 225.5 days (95% CI 200–271); *P < *0.01]. Elevated tumor markers have been used to aid in the diagnosis of hilar cholangiocarcinoma; the average CEA and CA 19‐9 concentrations at presentation were 12.0 ± 2.7 μg/L and 3236.4 ± 1189.7 U/mL*,* respectively (Table 3

**Table 3.** Laboratory characteristics at presentation

Laboratory marker	Value
Albumin*,* g/dL	3.2 ± 0.07
Total bilirubin*,* μmol/L	15.6 ± 0.9
Cancer antigen 19‐9*,* U/mL	3236.4 ± 1189.7
Carcinoembryonic antigen*,* μg/L	12.0 ± 2.7
Alkaline phosphatase*,* U/L	458.5 ± 33.7

Data presented as mean ± standard deviation

). The most frequent metastatic locations at presentation were the lymph nodes (23.3%; *n = *27).

## Discussion

Hilar cholangiocarcinoma is a devastating malignancy with a poor overall prognosis. There is a need for prognostic markers in patients who present with this condition. Previous studies on prognostic factors have been limited in their scope. The majority have assessed prognostic factors only in patients who underwent surgical resection; in these studies*,* pre-operative albumin*,* tumor grade/size*,* extent of resection*,* lymph node/vascular involvement, and elevated CEA/CA19‐9 tumor markers have been established as important prognostic factors [19–26]. The only study to assess variables in all patients diagnosed with hilar cholangiocarcinoma was also limited in the extent of data collected and analyzed. Laboratory values assessed on univariate analysis included bilirubin*,* alkaline phosphatase*,* GGT and leukocyte count, while multivariate analysis was not completed [27]. The present study included data from all patients diagnosed with hilar cholangiocarcinoma over a 10-year period, including assessment of demographic data*,* medical history/risk factors*,* and presenting laboratory data. It demonstrated that albumin level >3.0 g/dL is an independent predictor of overall survival. Factors such as CA19‐9 ≤ 200 U/mL*,* CEA ≤ 10 μg/L and a medical history without cirrhosis or diabetes may also be associated with increased overall survival*,* although these are not independent predictors of survival.

In early epidemiological studies*,* lower serum albumin concentrations were associated with an increased risk of cancer-related mortality; further*,* in gastrointestinal malignancies such as colorectal*,* gastric and hepatocellular carcinoma*,* low albumin has been found to be a poor prognostic indicator of survival [28–30]. In our study*,* a serum albumin >3.0 g/dL conferred an independent survival advantage with a significantly greater median length of survival (45 weeks longer; HR 0.31). Sub-group analysis assessed the impact of cirrhosis on lower albumin levels. Patients with underlying cirrhosis represented 6% of all patients in our data set and were associated with lower overall survival on univariate analysis; but the absence of cirrhosis was not found to be an independent predictor of survival; thus the relationship between low albumin and prognosis in hilar cholangiocarcinoma cannot be explained by cirrhosis alone. Cancer cachexia may provide an alternate explanation. Hypoalbuminemia is present in the setting of a systemic inflammatory response and is associated with weight loss [31, 32]. Weight loss prior to presentation occurred in 46% of our patients, with an average 24 pounds (10.89 kg) lost prior to evaluation. The interplay of hypoalbuminemia*,* cachexia and the inflammatory response may contribute to progressive loss of vital protein in patients with hilar cholangiocarcinoma; further, the long half-life of serum albumin may provide a longitudinal assessment of the patients’ wellbeing. As stated previously, studies have demonstrated that pre-operative albumin concentration is an independent predictor of survival after surgical resection—a finding that is now extended to the overall survival of all patients presenting with hilar cholangiocarcinoma, regardless of therapeutic management. Serum albumin is inexpensive and reproducible, and nearly all patients with biliary pathology or suspected hilar cholangiocarcinoma will have a hepatic panel measured.

Tumor markers have been evaluated as prognostics factors in patients undergoing surgical resection for cholangiocarcinoma—specifically CEA and CA 19‐9 [22*,* 33]. In our analysis*,* a CEA > 10 μg/L and a CA19‐9 > 200 U/mL were associated with diminished median overall survival by 17 weeks and 35 weeks*,* respectively. Although not independent predictors of survival*,* the data suggests that patients with higher CA19‐9 and CEA serum concentrations may have diminished survival.

The prevalence of PSC in cholangiocarcinoma ranges from 5–36% [34*,* 35]. Six patients (5.2%) were identified with PSC and hilar cholangiocarcinoma in this study. Previous data demonstrates that survival is significantly decreased in patients with PSC and cholangiocarcinoma. Given our limited sample set*,* one should be cautious when interpreting survival in patients with PSC and hilar cholangiocarcinoma from our data set.

There are limitations to our study. Although the sample set was fairly large (given the prevalence of disease)*,* the retrospective nature of the study limited data collection and analysis; further*,* during the decade-long study period, changes in therapeutic options may have occurred. Despite these limitations*,* this study has provided a comprehensive assessment of prognostic factors for overall survival in patients with hilar cholangiocarcinoma.

In summary*,* this study was the largest retrospective analysis of prognostic factors in all patients diagnosed with hilar cholangiocarcinoma at a single institution. Overall, hilar cholangiocarcinoma is associated with a poor prognosis and the data demonstrated that an albumin level >3.0 g/dL at presentation is an independent predictor of overall survival.

### Author contributions

Abhijeet Waghray: study concept and design; acquisition of data; analysis and interpretation of data; drafting of the manuscript; critical revision of the manuscript
Anastasia Sobotka: acquisition of data; drafting of the manuscript
Carlos Romero Marrero: critical revision of the manuscript
Bassam Estfan - critical revision of the manuscript
Federico Aucejo: critical revision of the manuscript
KV Narayanan Menon: study concept and design; analysis and interpretation of data; critical revision of the manuscript; study supervision

*Conflict of interest statement: * none declared.

## References

[1] Cai WK Sima H Chen BD Risk factors for hilar cholangiocarcinoma: a case-control study in China. World J Gastroenterol 2011;17:249–53. 21246000 10.3748/wjg.v17.i2.249 PMC3020381

[2] Klatskin G Adenocarcinoma of the Hepatic Duct at Its Bifurcation within the Porta Hepatis. An Unusual Tumor with Distinctive Clinical and Pathological Features. Am J Med 1965;38:241–56. 14256720 10.1016/0002-9343(65)90178-6

[3] Nakeeb A Pitt HA Sohn TA Cholangiocarcinoma. A spectrum of intrahepatic*,* perihilar*,* and distal tumors. Ann Surg 1996;224:463–75. 8857851 10.1097/00000658-199610000-00005 PMC1235406

[4] Burke EC Jarnagin WR Hochwald SN Hilar Cholangiocarcinoma: patterns of spread*,* the importance of hepatic resection for curative operation*,* and a presurgical clinical staging system. Ann Surg 1998;228:385–94. 9742921 10.1097/00000658-199809000-00011 PMC1191497

[5] Nagorney DM Donohue JH Farnell MB Outcomes after curative resections of cholangiocarcinoma. Arch Surg 1993;128:871–79. 8393652 10.1001/archsurg.1993.01420200045008

[6] Soares KC Kamel I Cosgrove DP Hilar cholangiocarcinoma: diagnosis*,* treatment options*,* and management. Hepatobiliary Surg Nutr 2014;3:18–34. 24696835 10.3978/j.issn.2304-3881.2014.02.05 PMC3955000

[7] Suarez-Munoz MA Fernandez-Aguilar JL Sanchez-Perez B Risk factors and classifications of hilar cholangiocarcinoma. World J Gastrointest Oncol 2013;5:132–38. 23919107 10.4251/wjgo.v5.i7.132 PMC3731526

[8] Tyson GL El-Serag HB Risk factors for cholangiocarcinoma. Hepatology 2011;54:173–84. 21488076 10.1002/hep.24351 PMC3125451

[9] Nuzzo G Giuliante F Ardito F Improvement in perioperative and long-term outcome after surgical treatment of hilar cholangiocarcinoma: results of an Italian multicenter analysis of 440 patients. Arch Surg 2012;147:26–34. 22250108 10.1001/archsurg.2011.771

[10] Cannon RM Brock G Buell JF Surgical resection for hilar cholangiocarcinoma: experience improves resectability. HPB (Oxford) 2012;14:142–49. 22221577 10.1111/j.1477-2574.2011.00419.x PMC3277058

[11] Saxena A Chua TC Chu FC Improved outcomes after aggressive surgical resection of hilar cholangiocarcinoma: a critical analysis of recurrence and survival. Am J Surg 2011; 202: 310–20. 21871986 10.1016/j.amjsurg.2010.08.041

[12] De Bellis M Sherman S Fogel EL Tissue sampling at ERCP in suspected malignant biliary strictures (Part 1). Gastrointest Endosc 2002;56:552–61. 12297773 10.1067/mge.2002.128132

[13] Fogel EL deBellis M McHenry L Effectiveness of a new long cytology brush in the evaluation of malignant biliary obstruction: a prospective study. Gastrointest Endosc 2006;63: 71–77. 16377319 10.1016/j.gie.2005.08.039

[14] Madariaga JR Iwatsuki S Todo S Liver resection for hilar and peripheral cholangiocarcinomas: a study of 62 cases. Ann Surg 1998;227:70–79. 9445113 10.1097/00000658-199801000-00011 PMC1191175

[15] Lygidakis NJ Singh G Bardaxoglou E Changing trends in the management of Klatskin tumor. Hepatogastroenterology 2004;51:689–96. 15143894

[16] Rea DJ Rosen CB Nagorney DM Transplantation for cholangiocarcinoma: when and for whom? Surg Oncol Clin n Am 2009;18:325–37, ix. 19306815 10.1016/j.soc.2008.12.008

[17] Lang H Sotiropoulos GC Kaiser GM The role of liver transplantation in the treatment of hilar cholangiocarcinoma. HPB (Oxford) 2005;7:268–72. 18333205 10.1080/13651820500372780 PMC2043099

[18] Berardi R Mocchegiani F Pierantoni C Resected biliary tract cancers: a novel clinical-pathological score correlates with global outcome. Dig Liver Dis 2013;45:70–74. 22999058 10.1016/j.dld.2012.08.012

[19] Ramacciato G Di Benedetto F Cautero N Prognostic factors and long term outcome after surgery for hilar cholangiocarcinoma: univariate and multivariate analysis. Chir Ital 2004;56:749–59. 15771027

[20] Ramacciato G Corigliano N Mercantini P [Prognostic factors after surgical resection for hilar cholangiocarcinoma]. Ann Chir 2006;131:379–85. 16806037 10.1016/j.anchir.2006.03.006

[21] Ramacciato G Nigri G Bellagamba R Univariate and multivariate analysis of prognostic factors in the surgical treatment of hilar cholangiocarcinoma. Am Surg 2010;76: 1260–68. 21140696

[22] Juntermanns B Radunz S Heuer M Tumor markers as a diagnostic key for hilar cholangiocarcinoma. Eur J Med Res 2010;15:357–61. 20947473 10.1186/2047-783X-15-8-357 PMC3458701

[23] Ruys AT Busch OR Rauws EA Prognostic impact of preoperative imaging parameters on resectability of hilar cholangiocarcinoma. HPB Surg 2013;2013:657309. 23861556 10.1155/2013/657309 PMC3687508

[24] Wirasorn K Ngamprasertchai T Chindaprasirt J Prognostic factors in resectable cholangiocarcinoma patients: Carcinoembryonic antigen*,* lymph node*,* surgical margin and chemotherapy. World J Gastrointest Oncol 2013;5:81–87. 23671735 10.4251/wjgo.v5.i4.81 PMC3648667

[25] Cai WK Lin JJ He GH Preoperative serum CA19‐9 levels is an independent prognostic factor in patients with resected hilar cholangiocarcinoma. Int J Clin Exp Pathol 2014;7:7890–98. 25550829 PMC4270533

[26] Regimbeau JM Fuks D Pessaux P Tumour size over 3 cm predicts poor short-term outcomes after major liver resection for hilar cholangiocarcinoma. By the HC-AFC-2009 group. HPB (Oxford) 2015;17:79–86. 24992279 10.1111/hpb.12296 PMC4266444

[27] Weber A Landrock S Schneider J Long-term outcome and prognostic factors of patients with hilar cholangiocarcinoma. World J Gastroenterol 2007;13:1422–26. 17457974 10.3748/wjg.v13.i9.1422 PMC4146927

[28] Gupta D Lis CG Pretreatment serum albumin as a predictor of cancer survival: a systematic review of the epidemiological literature. Nutr J 2010;9:69. 21176210 10.1186/1475-2891-9-69 PMC3019132

[29] Lai CC You JF Yeh CY Low preoperative serum albumin in colon cancer: a risk factor for poor outcome. Int J Colorectal Dis 2011;26:473–81. 21190025 10.1007/s00384-010-1113-4

[30] Knekt P Hakulinen T Leino A Serum albumin and colorectal cancer risk. Eur J Clin Nutr 2000;54:460–62. 10878646 10.1038/sj.ejcn.1600997

[31] Scott HR McMillan DC Forrest LM The systemic inflammatory response*,* weight loss*,* performance status and survival in patients with inoperable non-small cell lung cancer. Br J Cancer 2002;87:264–67. 12177792 10.1038/sj.bjc.6600466 PMC2364225

[32] Al-Shaiba R McMillan DC Angerson WJ The relationship between hypoalbuminaemia*,* tumour volume and the systemic inflammatory response in patients with colorectal liver metastases. Br J Cancer 2004;91:205–7. 15213726 10.1038/sj.bjc.6601886 PMC2409827

[33] Zong D Zeng Y The value of CA19‐9 and CEA in predicting resectability of hilar cholangiocarcinoma. Sichuan Da Xue Xue Bao Yi Xue Ban 2014;45:819–22. 25341348

[34] Miros M Kerlin P Walker N Predicting cholangiocarcinoma in patients with primary sclerosing cholangitis before transplantation. Gut 1991;32:1369–73. 1661259 10.1136/gut.32.11.1369 PMC1379170

[35] Helzberg JH Petersen JM Boyer JL Improved survival with primary sclerosing cholangitis. A review of clinicopathological features and comparison of symptomatic and asymptomatic patients. Gastroenterology 1987;92:1869–75. 3569762 10.1016/0016-5085(87)90618-4

